# Cystic echinococcosis in the Eastern Mediterranean region: Neglected and prevailing!

**DOI:** 10.1371/journal.pntd.0008114

**Published:** 2020-05-07

**Authors:** Mehdi Borhani, Saeid Fathi, Samia Lahmar, Haroon Ahmed, Mohanad Faris Abdulhameed, Majid Fasihi Harandi

**Affiliations:** 1 Research Center for Hydatid Disease in Iran, School of Medicine, Kerman University of Medical Sciences, Kerman, Iran; 2 Department of Parasitology, Faculty of Veterinary Medicine, University of Tehran, Tehran, Iran; 3 Parasitology Laboratory, National School of Veterinary Medicine, Sidi Thabet, Tunisia; 4 Department of Biosciences, COMSATS University Islamabad (CUI), Islamabad, Pakistan; 5 College of Veterinary Medicine, University of Basrah, Basrah, Iraq; Istituto Superiore di Sanità, ITALY

## Abstract

Cystic echinococcosis (CE) is distributed worldwide, extending from China to the Middle East and from Mediterranean countries to the sub-Saharan Africa and South America. According to WHO, one million people around the world are suffering from CE with an estimated burden of 183,573 DALYs. The annual monetary burden of the disease due to treatment costs and CE-related livestock losses has been estimated at US$ 3 billion. CE is endemic in all countries within the WHO Eastern Mediterranean Regional Office (EMRO). The region, which includes most of the Middle East and North Africa, is one of the most ancient foci of the domestic cycle of CE and is recognized as one of the major hotspots of CE. There are 22 countries in the EMRO, where about 688 million people are living at risk of CE. In many EMRO countries, little is known about CE epidemiology and transmission. WHO included echinococcosis in a list of 17 neglected tropical diseases (NTDs) and 12 neglected zoonotic diseases (NZDs). Accordingly, different regional offices of WHO organized several initiatives for CE control and prevention. WHO’s Western Pacific regional office considered echinococcosis as one of the region’s major health topics, and several preventive measures have been implemented in the American region with the support of Pan American Health Organization (PAHO) in Argentina, Peru, Uruguay, and Chile. Although CE is endemic in all 22 EMRO countries, surprisingly, CE is absent from the health topics list of diseases and conditions in this region. Therefore, CE clearly requires further attention in the WHO EMRO agenda, and the need for elaboration of specific measures for CE control is becoming apparent in EMRO countries, where substantial collaborations among the member states and WHO EMRO is of paramount importance. Major topics of collaborative activities include training programs and health communication on different aspects of CE control, analysis of CE burden, national and international surveillance and disease registry systems, technical support to promote epidemiological studies for collecting baseline data, cost–benefit analysis of control interventions, and intersectoral cooperation among the agriculture, veterinary, medical, and health sectors.

Cystic echinococcosis (CE) is a major parasitic disease of humans and animals caused by the small intestinal tapeworm of carnivores, *Echinococcus granulosus*. CE is distributed worldwide, extending from China and Central Asia to the Middle East and Mediterranean countries (Southern Europe, North Africa, and Western Asia) as well as sub-Saharan Africa and South America [1].

According to WHO, 1 million people around the world are suffering from CE, and an estimated burden of 183,573 (88,082–1,590,846) disability-adjusted life years (DALYs) has been attributed to this zoonotic infection [2]. The annual monetary burden of the disease due to treatment costs and CE-related livestock losses has been estimated at US$3 billion [3].

WHO Eastern Mediterranean Region (EMRO), which includes most of the Middle East and North Africa (MENA), is one of the most ancient foci of the domestic cycle of CE and is recognized as one of the major hotspots of CE [4,5]. The stable endemicity of CE in this region is largely associated with several factors, including diverse intermediate host species (sheep, goats, camels, cattle, buffalos, and wild pigs), traditional farming and livestock husbandry, poor quality and/or unregulated abattoirs, widespread practice of home slaughter, high consumption of raw vegetables, large numbers of stray dogs, and low level of public awareness about CE transmission [1,6,7]. CE imposes substantial costs to low- and middle-income countries that is estimated at 0.01% to 0.04% of nation’s gross domestic product (GDP) [8].

There are 22 countries in EMRO, where about 688 million people are living at risk of CE. Actually, CE is endemic in all EMRO countries. In many EMRO countries, little is known about CE epidemiology and transmission. For instance, although CE is endemic in Pakistan, the country has been neglected in international collaborations on echinococcosis control, largely due to the paucity of information in the country [9]. There are no recent data on CE status in almost half of the EMRO member states, where comprehensive epidemiological data are undoubtedly essential for CE control programs.

WHO included echinococcosis in a list of 17 neglected tropical diseases (NTDs) and 12 neglected zoonotic diseases (NZDs) [10,11]. Accordingly, different regional offices of WHO organized several initiatives for CE control and prevention. WHO’s Western Pacific regional office considered echinococcosis as one of the region’s major health topics, while CE is currently endemic in 2 out of 37 member countries (China and Mongolia). CE has been increasingly reported in the WHO European Region (EURO) due to the recent influx of refugees from the MENA region to Europe [12]. Particular attention has been made to the foodborne diseases in Europe and echinococcosis was considered by WHO EURO as an important food safety concern. Considerable burden is imposed to the food industry because of foodborne infections, including CE. WHO EURO highlighted food and nutrition policies and steps necessary for decision makers to reduce the burden of foodborne disease and mortality in Europe [13,14].

CE as a major neglected infectious disease has received special attention in the Americas through the “South American Initiative for the Control and Surveillance of Cystic Echinococcosis /Hydatidosis”. Several preventive measures have been implemented in the region with the support of PAHO in Argentina, Peru, Uruguay, and China [15].

WHO EMRO has announced plans for training, monitoring, and epidemiological evaluation of several NTDs, including lymphatic filariasis, trachoma, leishmaniasis, schistosomiasis, and soil-transmitted helminths [16]. Although CE is endemic in all 22 EMRO countries, surprisingly, CE is absent from the health topics list of diseases and conditions in this region. Therefore CE clearly requires further attention in the WHO EMRO agenda to alert policy makers to this particular concern. Thereby, the need for elaboration of specific measures for CE control is becoming apparent in EMRO countries, where substantial collaborations among the member states and WHO EMRO is of paramount importance.

Major topics of collaborative activities include training programs and health communication on different aspects of CE control, analysis of CE burden, national and international surveillance and disease registry systems, technical support to promote epidemiological studies for collecting baseline data, cost–benefit analysis of control interventions, and intersectoral cooperation among the agriculture, veterinary, medical, and health sectors.

As part of the new roadmap on NTDs, WHO would coordinate and support member states to develop intervention programs for the prevention and control of NTDS, including cystic and alveolar echinococcosis. Initiatives need to be started by EMRO and its members through practical participatory planning to mobilize resources for incorporating echinococcosis and other human zoonotic diseases into the One Health domains.
